# Specificity and sensitivity of the SeLECT score in predicting late seizures in patients undergoing intravenous thrombolytic treatment and the effect of diabetes mellitus and leukoaraiosis

**DOI:** 10.1055/s-0043-1767764

**Published:** 2023-04-14

**Authors:** Yasemin Dinç, Aylin Bican Demir, Güven Özkaya, Mustafa Bakar

**Affiliations:** 1 Uludağ University, Faculty of Medicine, Department of Neurology, Bursa, Türkiye.; 2 Bursa Uludag University, Faculty of Medicine, Department of Biostatistics, Bursa, Türkiye.

**Keywords:** Stroke, Seizures, Intravenous Administration, Tissue Plasminogen Activator, Acidente Vascular Cerebral, Convulsões, Administração Intravenosa, Ativador de Plasminogênio Tecidual

## Abstract

**Background** Seizures after stroke can negatively affect the prognosis of ischemic stroke and cause a decrease in quality of life. The efficacy of intravenous (IV) recombinant tissue plasminogen activator (rt-PA) treatment in acute ischemic stroke has been demonstrated in many studies, and IV rt-PA treatment has been increasingly used around the world. The SeLECT score is a useful score for the prediction of late seizures after stroke and includes the severity of stroke (Se), large artery atherosclerosis (L), early seizure (E), cortical involvement (C), and the territory of the middle cerebral artery (T). However, the specificity and sensitivity of the SeLECT score have not been studied in acute ischemic stroke patients that received IV rt-PA treatment.

**Objective** In the present study, we aimed to validate and develop the SeLECT score in acute ischemic stroke patients receiving IV rt-PA treatment.

**Methods** The present study included 157 patients who received IV thrombolytic treatment in our third-stage hospital. The 1-year seizure rates of the patients were detected. SeLECT scores were calculated.

**Results** In our study, we found that the SeLECT score had low sensitivity but high specificity for predicting the likelihood of late seizure after stroke in patients administered IV rt-PA therapy. In addition to the SeLECT score, we found that the specificity and sensitivity were higher when we evaluated diabetes mellitus (DM) and leukoaraiosis.

**Conclusion** We found that DM was an independent risk factor for late seizures after stroke in a patient group receiving thrombolytic therapy, and late seizures after stroke were less frequent in patients with leukoaraiosis.

## Resumo

**Antecedentes** As convulsões após o AVC podem afetar negativamente o prognóstico do AVC isquêmico e causar uma diminuição na qualidade de vida. A eficácia do tratamento com ativador do plasminogênio tecidual recombinante (rt-PA) intravenoso (IV) no AVC isquêmico agudo foi demonstrada em muitos estudos, e o tratamento com rt-PA IV tem sido cada vez mais usado em todo o mundo. A pontuação SeLECT é uma pontuação útil para a previsão de convulsões tardias após AVC e inclui a gravidade do AVC (Se), aterosclerose de grandes artérias (L), convulsão precoce (E), envolvimento cortical (C) e o território do meio artéria cerebral (T). No entanto, a especificidade e a sensibilidade do escore SeLECT não foram estudadas em pacientes com AVC isquêmico agudo que receberam tratamento IV com rt-PA.

**Objetivo** No presente estudo, objetivamos validar e desenvolver o escore SeLECT em pacientes com AVC isquêmico agudo recebendo tratamento IV com rt-PA.

**Métodos** O presente estudo incluiu 157 pacientes que receberam tratamento trombolítico IV em nosso hospital de terceiro estágio. As taxas de convulsão de 1 ano dos pacientes foram detectadas. Os escores SeLECT foram calculados.

**Resultados** Em nosso estudo, descobrimos que o escore SeLECT apresentou baixa sensibilidade, mas alta especificidade para prever a probabilidade de convulsão tardia após AVC em pacientes que receberam terapia IV com rt-PA. Além do escore SeLECT, descobrimos que a especificidade e a sensibilidade foram maiores quando avaliamos diabetes mellitus (DM) e leucoaraiose.

**Conclusão** Descobrimos que DM foi um fator de risco independente para convulsões tardias após AVC em um grupo de pacientes recebendo terapia trombolítica, e convulsões tardias após AVC foram menos frequentes em pacientes com leucoaraiose.

## INTRODUCTION

Acute ischemic stroke is a block of cerebral blood circulation to an area of the brain, typically in a vascular territory, resulting in a corresponding loss of neurologic function. Acute ischemic stroke is one of the major causes of disability and death in the world, affecting 1 in 6 adults, with ∼ 3 to 6 million cases of stroke per year. 1

Patients with ischemic stroke have an increased risk of seizures, with stroke being the leading cause of seizures in adults. 2 Seizures after stroke could negatively affect the prognosis of the stroke and cause a decrease in quality of life. 3 4 5

The efficacy of intravenous (IV) recombinant tissue plasminogen activator (rt-PA) treatment for acute ischemic stroke has been demonstrated in many studies, and IV rt-PA treatment for acute ischemic stroke has been increasingly used around the world. 6 7

There is no consensus on how IV rt-PA treatment affects seizures after acute ischemic stroke. Animal and in vitro experiments have shown that rt-PA is cytotoxic. 8 9 10 11 Some studies have reported that IV rt-PA treatment increases rates of seizures after stroke; however, epileptic seizures have not been reported in phase studies of IV rt-PA treatment. 6 7 12 13 14

Some authors have described that IV rt-PA treatment for adult acute stroke patients reduces the risk of late seizures after stroke due to possible recanalisation. 15 Seizures after stroke can occur early (≤ 7 days after the onset of stroke) or late (> 7 days). 16 In accordance with the current International League Against Epilepsy (ILAE) definition, a single late seizure after a stroke qualifies as structural epilepsy by increasing (> 60%) the risk of frequency within the next 10 years. 17

The SeLECT score is useful for the prediction of late seizures after a stroke. Its specificity and sensitivity have been determined in previous validation studies. 18 As shown in Table 1 , the SeLECT score includes (Se) severity of the stroke, (L) large artery atherosclerosis, (E) early seizure, (C) cortical involvement, and (T) territory of the middle cerebral artery (MCA).

**Table 1:** SeLECT score predictors and points

SeLECT score (points)		
(SE)Severity of stroke	NIHSS < 3	0
	NIHSS 4–10	1
	NIHSS > 11	2
(L)Large artery atherosclerosis	No	0
	Yes	1
(E)Early seizures	No	0
	Yes	3
(C)Cortical involvement	No	0
	Yes	2
(T)Territory of the MCA	No	0
	Yes	1

The specificity and sensitivity of the SeLECT score are not known in the acute ischemic stroke patient group that received IV rt-PA treatment. In the present study, we aimed to validate and develop the SeLECT score in acute ischemic stroke patients receiving IV rt-PA therapy.

## METHODS

We retrospectively included 157 patients who were diagnosed with acute ischemic stroke at the Uludağ University medical faculty emergency department and were treated with IV rt-PA between January 1, 2020 and January 1, 2021.

Approval for the present study was obtained from the clinical research ethics committee of Uludağ University, Faculty of Medicine, with the decision number 2022-2/11, dated January 19, 2022. Because it was a retrospective study, patient consent was not required.

The inclusion criteria for the study were as follows: patients receiving IV rt-PA treatment in the neurology department of the Faculty of Medicine of the Uludağ University after a diagnosis of acute ischemic stroke, patients' stroke etiology being clarified, patients receiving regular follow-ups in the Uludağ University Faculty of Medicine's stroke outpatient clinic for a year.

The exclusion criteria for the study were as follows: patients having an epilepsy diagnosis prior to their stroke, taking medicine that affects the epileptic threshold (anti-seizure medication), having malignancy, and having a life expectancy shorter than a year.

Between the specified dates, 198 patients received IV rt-PA treatment in the neurology department of the Faculty of Medicine of Uludağ University. Forty-one patients were excluded from the study. Twenty-eight patients were excluded because they died before the completion of 1 year. Four patients were excluded because they were diagnosed with pre-stroke epilepsy. Nine patients were excluded because they were using drugs that alter the epileptic threshold.

Patients diagnosed with ischemic stroke after neuroimaging in the emergency department were examined by a neurologist. The National Institutes of Health Stroke Scales (NIHSS) were calculated and recorded during the epicrisis. In the emergency room, computed tomography (CT) angiography was performed together with brain CT.

Early ischemic changes and Alberta Stroke Program Early CT (ASPECT) scores were evaluated from the brain CT.

Considering the indications and contraindications in the stroke guidelines, IV thrombolytic therapy was applied. 19 The presence of major vessel occlusion in the CT angiography was also evaluated. Mechanical thrombectomy was performed in patients with a pre-stroke modified Rankin score (mRs) < 2 and major vessel occlusion. 19

Intravenous thrombolytic therapy was applied at a dose of 0.9 mg/kg, and all patients were followed for at least 7 days in the neurology clinic and at least 1 year in the neurology outpatient clinic after therapy.

Brain CT scans of the patients were performed in the emergency department just before the IV thrombolytic treatment, 24 hours after the treatment, and immediately in the instance of neurological deterioration.

Patients' symptom onset time, NIHSS score, ASPECT score, presence of hypertension (HT) in their history, previous history of DM, presence of atrial fibrillation, stroke etiology, hemoglobin value, creatinine value, and serum low-density lipoprotein value were recorded in the epicrisis.

HBA1c and fasting and postprandial serum glucose levels of all patients were tested. Patients with serum HBA1c above 6.5 mmol/mol, patients with fasting blood glucose above 125 mmol/L twice, and those whose serum glucose level measured above 200 mmol/L at any time were considered diabetic.

The stroke aetiology of the patients was determined by a stroke neurologist using the Trial of ORG 10172 in acute stroke treatment (TOAST) stroke classification.

Early neurological deterioration was defined as a two-point increase in the NIHSS score in the first 72 hours after hospitalisation. 20 Symptomatic intracranial hemorrhage was defined as intracranial hemorrhage leading to death or neurological worsening of an NIHSS score ≥ 4 from baseline within 22 to 36 hours of treatment. 21 To measure the severity of leukoaraiosis, white matter hypodensities in the anterior and posterior horns of the lateral ventricle were evaluated in axial brain CT. 22 Leukoaraiosis was evaluated blindly by a neuroradiologist using the first cranial CT performed in the emergency room.

The clinical outcomes of the patients were evaluated in the neurology outpatient clinic in the 3 ^rd^ month. Those with mRs scores of 0, 1, and 2 were evaluated as having good clinical outcomes, and those with scores of 3 to 6 as having poor clinical outcomes.

The description of early and late seizures was made according to the ILAE criteria. A patient who had a seizure in the 1 ^st^ week was evaluated as having had an early seizure after stroke, and a patient who had a seizure after the 7 ^th^ day was evaluated as having had a late seizure after stroke. 16

The SeLECT score includes five predictors: the severity of the stroke, early seizures, large artery atherosclerosis, cortical involvement, and territory of the MCA.

The SeLECT scores of the patients were calculated according to the final diffusion cranial magnetic resonance imaging (MRI) and neurological examination performed at discharge.

As shown in Table 1 , the highest SeLECT value is 9 points, and the lowest is 0 points. The predictor of the SeLECT score has got different points. All patients were evaluated regularly every month in the neurology outpatient clinic and questioned as to whether they had seizures or not.

An electroencephalography (EEG) was performed for all patients in the 1 ^st^ week, the 3 ^rd^ month, and 1 ^st^ year of IV rt-PA treatment. Patients were diagnosed with epilepsy based on clinical and EEG findings. Records were taken every month as to whether the patients who were evaluated clinically in the neurology outpatient clinic had epileptic seizures.

### Statistical analysis

The clinical, demographic, and radiological information and data were compared regarding whether patients treated with IV rt-PA had late seizures after stroke or not.

Statistical analysis was implemented using IBM SPSS Statistics for Windows version 23.0 (IBM Corp., Armonk, New York, USA) and MedCalc Statistical Software version 19.1.5 (MedCalc Software, Ostend, Belgium).

The Shapiro-Wilk test was conducted to determine whether the data presented normal distribution.

The means and standard deviations (SDs) or medians (25–75% quartiles) were given for the analysis of continuous variables. Frequencies and percentages were used for categorical variables. Either a two-sided Mann–Whitney U test or a two-sided independent sample t-test was implemented to compare the differences between groups for continuous variables.

A two-sided Fisher's exact and Pearson chi-squared test for categorical variables were applied to compare the differences between the groups. Binary logistic regression was performed, and the crude odds ratios (ORs), along with their 95% confidence intervals (CIs), were reported. Multivariable binary logistic regression analysis was implemented, and the adjusted ORs and 95%CIs were calculated. A *p* -value < 0.05 was considered significant. The receiver operating characteristic (ROC) curve was applied for evaluation of the cutoff value, sensitivity, and specificity of the parameter for predicting late seizures after stroke. The area under the ROC curve (AUC) was used for the comparison of models in the evaluation of late seizures after stroke.

## RESULTS

A total of 157 patients, 92 (58.60%) male, and 65 (41.40%) females, were included in the present study. The mean age of women was 70.72 ± 11.91 years old, and the mean age of men was 69.10 ± 12.98 years old.

The mean age of women and men was statistically similar ( *p* > 0.05). The stroke etiology of each patient was classified using TOAST stroke classification and the stroke etiology was determined in 85 (54.4%) patients as cardioembolism, in 26 as large artery atherosclerosis (16.56%), in 8 as small vessel occlusion (5.1%), in 3 as other determined etiologies (1.91%), and in 35 as undetermined (22.29%).

IV thrombolytic therapy was administered to all patients, and the symptom treatment time was 190.41 ± 50.23 minutes on average.

Mechanical thrombectomy was performed in 49 (31.21%) patients, and the mean symptom-needle time of the patients was 245.56 ± 55.24 minutes.

Hemorrhagic transformation was found in 19 (12.10%) of the patients, 6 (3.82%) had type 1 hemorrhagic transformation, 7 (4.45%) had type 2 hemorrhagic transformation, 3 (1.91%) hemorrhagic transformations were found to be type 1 parenchymal hematomas, and 3 (1.91%) were type 2 parenchymal hematomas. Nine (5.32%) patients had symptomatic intracranial hemorrhage. While 95 (60.5%) patients had good clinical outcomes, 62 (39.5%) patients had poor clinical outcomes.

Fourteen (8.9%) of the patients had early seizure after stroke. Anti-seizure medication was started in these patients. Apart from these, prophylactic anti-seizure medication was started in none of the patients. Nineteen (12.1%) of the patients had a late seizure after stroke.

A significant statistical difference was detected between stroke patients with and without late seizures after stroke in terms of the presence of DM ( *p* = 0.006), presence of coronary artery disease ( *p* = 0.015), NIHSS value in the emergency room ( *p* = 0.004), NIHSS value at discharge ( *p* = 0.006), clinical outcome ( *p* = 0.024), severity of leukoaraiosis ( *p* = 0.005), SeLECT score ( *p* < 0.001), early seizures ( *p* < 0.001), cortical involvement ( *p* = 0.009), and symptomatic intracranial hemorrhage ( *p* = 0.001).

In contrast, there was no significant difference between the groups in term of age ( *p* = 0.511), gender ( *p* = 0.417), presence of atrial fibrillation ( *p* = 0.156), being a smoker ( *p* = 0.456), systolic blood pressure value ( *p* = 0.116), ASPECT score ( *p* = 0.155), presence of major vessel occlusion ( *p* = 0.143), presence of stroke due to cardioembolism ( *p* = 0.437), presence of stroke due to large artery occlusion ( *p* = 0.108), and involvement of the MCA ( *p* = 0.229) ( Table 2 ).

**Table 2:** Comparison of clinical, demographic, and radiological features of the patients with and without late seizure after stroke

Variables	Patients having late seizure after stroke ( *n* = 19)	Patients not having late seizure after stroke ( *n* = 138)	*p-* value
Age (years old) (mean ± SD)*	69.15 ± 8.13	69.86 ± 13.04	0.511
Sex (male gender)**	12 (63.15%)	80 (57.97%)	0.434
Hypertension**	17 (89.47%)	101 (73.18%)	0.099
Diabetes mellitus**	13 (68.42%)	48 (34.78%)	**0.006**
Coronary artery disease**	15 (78.94%)	69 (50%)	**0.015**
Atrial fibrillation**	12 (63.13%)	66 (47.82%)	0.154
Being smoker **	9 (47.36%)	60 (43.47%)	0.466
NIHSS value before IV thrombolitic treatment (mean ± SD)*	20.73 ± 7.04	15.34 ± 7.29	**0.004**
NIHSS value at discharge (mean ± SD)*	17.52 ± 11.06	9.54 ± 9.85	**0.006**
Blood pressure systolic value (mean ± SD)*	156.57 ± 21.21	152.03 ± 24.56	0.316
Blood pressure diastolic value (mean ± SD)*	93.47 ± 15.06	87.41 ± 14.71	0.116
Glucose (mg/dl), (mean ± SD)*	159.15 ± 75.08	140.71 ± 56.30	0.093
Creatinine (mg/dl), (mean ± SD)*	0.94 ± 0.26	1.04 ± 0.70	0.823
Clinical outcome**	7 (36.84%)	88 (63.76%)	**0.024**
Early neurological deterioration **	8 (42.10%)	24 (17.39%)	**0.018**
ASPECT (mean ± SD)*	8.15 ± 2.03	8.92 ± 1.32	0.155
Leukoaraiosis (mean ± SD)*	0.21 ± 0.91	2.17 ± 3.11	**0.005**
Presence of major vessel** occlusion	12 (63.15%)	65 (47.10%)	0.143
Mechanical thrombectomy**	4 (21.05%)	45 (32.60%)	0.269
Stroke due to cardioembolism**	11 (57.89%)	73 (52.89%)	0.437
Stroke due to large artery** atherosclerosis	5 (26.31%)	22 (15.94%)	0.206
Semptom-treatment time (IV rt-PA treatment), (mean ± SD)*	200.00 ± 53.74	189.09 ± 49.79	0.277
SeLECT score (mean ± SD)*	6.84 ± 1.89	3.46 ± 1.57	**< 0.001**
Early seizures**	14 (73.68%)	0 (0%)	**< 0.001**
Cortical involvement**	15 (78.94%)	66 (47.82%)	**0.009**
Territory of the MCA**	15 (78.94%)	93 (67.39%)	0.229
Symptomatic intracerebral hemorrhages**	5 (26.31%)	4 (2.89%)	**0.001**

Abbreviations: ASPECT, Alberta stroke programme early CT score; IV, intra-venous; MCA, middle cerebral artery; rt-PA, tissue plasminogen activator; SD, standard deviation.

Notes: Significant variables are shown in bold. *Mann-Witney U test, **Pearson chi-squared test/continuity correction test/Fisher Exact test.

Variables associated with late seizure after stroke were evaluated by univariate binary logistic regression ( Table 3 ). For the variables found to be significant, backward stepwise multivariate binary logistic regression analysis was performed. The analysis indicated that the SeLECT score *(p* < 0.001; OR = 4.435), presence of DM ( *p* = 0.014; OR = 9.105), and severity of leukoaraiosis ( *p* = 0.068; OR = 0.628) variables associated with late seizure after stroke were statistically significant ( Table 4 ).

**Table 3:** Evaluation of significant variables using univariate binary logistic regression

Variables	95%CI for OR			
	*p* -value	OR	Lower	Upper
Diabetes mellitus	**0.008**	4.062	1.452	11.366
Coronary artery disease	**0.025**	3.750	1.185	11.871
Blood pressure diastolic value	0.093	1.027	0.996	1.059
Clinical outcome	**0.030**	3.017	1.116	8.158
Early neurological deterioration	**0.016**	3.455	1.256	9.499
Aspect	**0.036**	0.728	0.541	0.979
Leukoariosis	**0.042**	0.608	0.376	0.983
SeLECT score	**< 0.001**	4.113	2.200	7.691
Symptomatic intracerebral hemorrhages	**0.001**	11.964	2.876	49.765

Abbreviation: CI, confidence interval; OR, odds ratio.

Notes: Significant variables are shown in bold; Significance of the model: *p* < 0.001.

**Table 4:** Evaluation of significant variables using multivariate binary logistic regression

Variables	95%CI for OR			
	*p* -value	OR	Lower	Upper
Diabetes mellitus	0.014	9.105	1.575	52.639
Leukoaraiosis	0.068	0.628	0.381	1.036
SeLECT score	< 0.001	4.435	2.032	9.678

Abbreviation: CI, confidence interval; OR, odds ratio.

Notes: Significant variables are shown in bold; Significance of the model: *p* < 0.001.

Two models were created: Model 1 and Model 2. Model 1 contained the total of SeLECT scores of the patients. Model 2 consisted of the SeLECT scores and the presence of DM and severity of leukoaraiosis.

**Figure 1:**
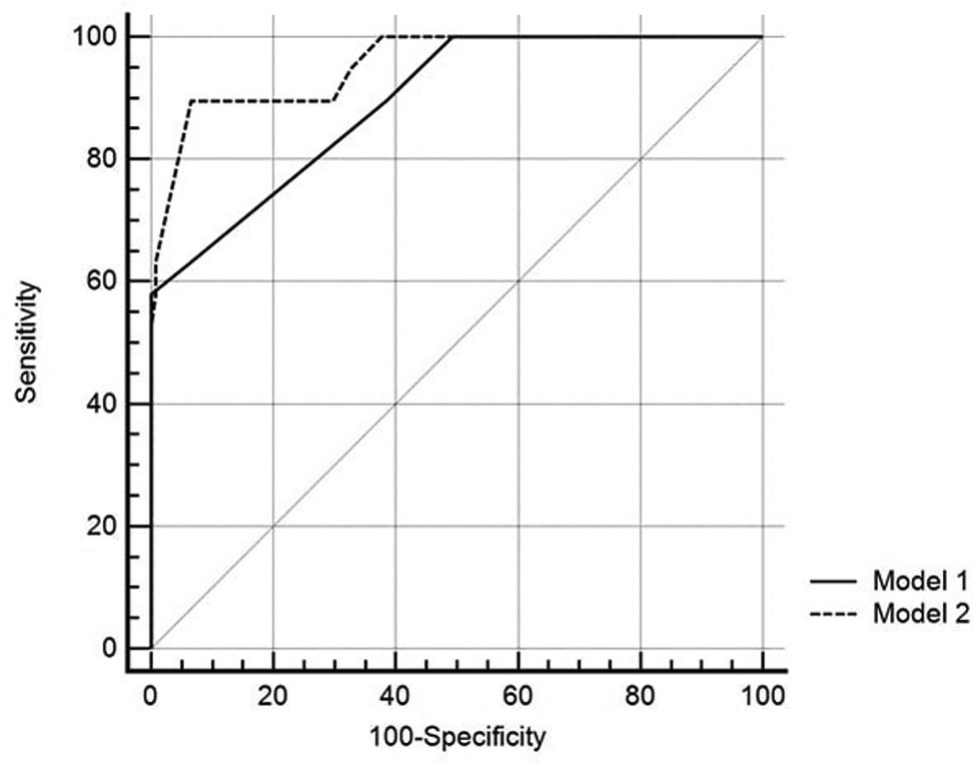
Comparison of area under the ROC curves (AUC) for models ( *p* = 0.013).

Table 5 and Figure 1 show the AUC, cutoff value, sensitivity, and specificity values for the model in which the variables were found to be significant only after the SeLECT score and multivariate binary logistic regression analyses were included.

**Table 5:** Diagnostic characteristics of models for late seizure after stroke

Model	AUC	*p* -value	Cutoff	Sensitivity (95%CI)	Specifity (95%CI)	Comparison of AUCs *p-* value
1: SeLECT Score	0.893	< 0.001	> 6	57.89 (33.5–79.7)	100.00 (97.4–100)	**0.013**
2: SeLECT score + DM + Leukoaraiosis	0.955	< 0.001	> 0.208	89.47 (66.9–98.7)	93.48 (88.0–97.0)	

Note: Significant variables are shown in bold; Significance of the model: p < 0.001.

A statistically significant difference was found between Model 1, which included only the SeLECT variable, and Model 2, which included the SeLECT score, presence of DM, and severity of leukoaraiosis variables, in terms of AUCs ( *p* = 0.013). The AUC value found for Model 2 (0.955) was higher than that found for Model 1 (0.893) ( Figure 1 ).

The sensitivity value for Model 2 was found to be 89.47 (66.9–98.7) and the specificity value of Model 2 was found to be 93.48 (88.0–97.0) ( Table 5 ).

## DISCUSSION

We found that DM was an independent risk factor for late seizure after stroke in patients receiving thrombolytic therapy. In our study, we found that the SeLECT score had low sensitivity but high specificity for estimating late seizure after stroke in patients administered IV rt-PA therapy according to the validation study. 18 According to another study that validated the SeLECT score, the SeLECT score of our patient population had lower specificity and higher sensitivity and the score's cutoff value we found in our study was 6, while the cutoff value in the present study was 4. 23

We determined that the presence of DM is an independent risk factor in these patients, while late seizures were less frequent in patients with leukoaraiosis. Therefore, in addition to the SeLECT score, the specificity and sensitivity of the presence of DM and leukoaraiosis are much higher.

The SeLECT score was successful in predicting late seizures after stroke in the validation study. 18 In our study, statistically significant results were found between early seizures, cortical involvement and stroke severity, and late seizure after stroke. By contrast, no statistically significant results were obtained regarding stroke due to large atherosclerosis and involvement of the MCA.

The possible reason no statistically significant correlation was found between stroke due to large artery atherosclerosis, the territory of the MCA, and late seizures after stroke could be that most of the patients who received IV thrombolytic therapy had cardioembolic strokes and involvement of the MCA. The most noticeable results of our study were for DM and leukoaraiosis, which were independent significant predictors of seizure after stroke in the patient group that we treated with IV rt-PA.

The presence of DM was found to be an independent risk factor for seizures after stroke in many previous studies. 24 25 However, the mechanism of the seizures occurring after stroke in diabetic patients has not yet been clarified.

Experimental studies have also shown that epileptogenesis is caused by hyperglycemia during ischaemia. 26 In addition to hyperglycemia, hypoglycemia is a frequent occurrence in diabetic patients. These metabolic derangements modify the balance between excitation and inhibition of neural networks. 27 28

Diabetes mellitus is also known to increase inflammation in the ischemic brain. Increasing evidence in recent years suggest that inflammatory and immune processes play a role in epileptogenesis. The inflammatory reaction caused by stroke could cause both early and late seizures. 29

Diabetes mellitus may also cause leukoaraiosis as a cause of subcortical lesions, but in our findings, DM was found to be an independent risk factor and is independent of leukoaraiosis.

Sestrin 3 (SESN3) is known to be a regulator of a proconvulsant gene network in the human epileptic hippocampus, and the risk of seizures is decreased by inhibition of high glucose metabolism rates via lactate dehydrogenase. 30 31 Sestrin 3 may play a role in the regulation of multiple pathways comprising the activated protein kinase (AMPK) mechanistic target of rapamycin complex 1 (mTORC1), and mechanistic target of rapamycin complex 2 (mTORC2) axes, which regulate hepatic insulin signaling and glucose metabolism. Studies conducted in diabetic animal models have indicated that the upregulation of sestrins in the hippocampus and SESN3 are associated with seizures after stroke in diabetic animals. 32 33

The pathophysiologies of early and late seizures after stroke are different. In contrast to early seizures after stroke, late seizures after stroke are a result of the development of gliosis and meningocerebral scarring. 34 Selective neuronal loss, changes in membrane properties, deafferentation, and collateral sprouting can bring about hyperexcitability and neuronal synchrony and cause seizures. 35 36

Leukoaraiosis is a radiologic finding that indicates the areas of hypoattenuation of the subcortical brain white matter on CT, and leukoaraiosis is usually seen as symmetrical. 37 The clinical importance of leukoaraiosis is not fully known. With ageing, arteries lose elasticity due to the accumulation of atherosclerotic plaques, amyloid, and hyalinization, which leads to ischemia and gliosis with consequent neurotransmission disorders. 38

Leukoaraiosis could be associated with advanced age, microinfarcts, and HT. 39 Although leukoaraiosis is known to be a disease of white matter, cortical volume reduction has been shown in studies performed in brains with leukoaraiosis. 40

In our study, we determined that late seizures after stroke were less frequent in patients with leukoaraiosis. To our knowledge, no previous study has evaluated leukoaraiosis and late seizures in patients treated with IV rt-PA. Leukoaraiosis also increases the risk of symptomatic intracranial hemorrhage in patients receiving IV thrombolytic therapy. 41

The fact that late seizures were less frequent in patients with leukoaraiosis is a surprising result of our study. The pathological studies suggest that leukoaraiosis is one manifestation of cerebral small vessel disease. This is supported by strong pathological and clinical associations with the other major manifestation of small vessel disease—lacunar stroke. 42 Since cortical lesions do not occur, the risk of poststroke seizure is low in ischemic stroke due to small vessel disease. 15 The relationship between leukoaraiosis and small vessel disease may explain this relationship.

Another possible reason for late seizures being less frequent in patients with leukoaraiosis may be the lower development of collateral sprouting neuronal synchrony in the leukoaraiosis brain tissue.

### Limitation of the present study

The greatest limitation of our study is that we used a single center and a limited sample size. Moreover, the present study was retrospective; the patients were evaluated only according to their medical records in our tertiary center.

In conclusion, in the present study, we found that the SeLECT score had high specificity but low sensitivity in predicting late seizures after stroke. In addition, we found that DM was an independent risk factor for late seizures after stroke in patients receiving thrombolytic therapy, and late seizures after stroke were less frequent in patients with leukoaraiosis. In addition to the SeLECT score, we found that the specificity and sensitivity were higher when we evaluated the severity of DM and leukoaraiosis. Clearer information could be obtained with multicenter prospective studies.
